# Abstract

**Published:** 1876-03

**Authors:** DeSaussure Ford, J. T. Johnson

**Affiliations:** President; Secretary


### ABSTRACT.

Dr. J. C. Reeve, in *The Clinic*, advocates the superior safety of the anæsthetic mixture of alcohol, chloroform, and ether, in the proportions of 1, 2, 3. He regards it every way preferable to either chloroform or ether.

Dr. Stoddard reports, in the *Detroit Review of Medicine and Pharmacy*, a remarkable case of missed labor. The pregnancy occurred thirteen months prior to death; at her expected time labor pains came on feebly; the physician administered an opiate, which quieted all pain. After the lapse of "a long time" he was again called; the pains were now even more efficient than before. After waiting some time and finding the pains dying away, ergot was administered in increasing doses without effect. A week or ten days after the use of the ergot, the patient had a chill, and from this time forward the fœtal movements ceased. In course of time numerous abscesses formed and were opened upon the surface of the abdomen, and discharged large quantities of offensive pus, which was followed by fœtid sanies and septic poisoning until the death of the mother. At the autopsy the uterus was found extensively adherent to the abdominal wall, and filled with the partially decayed remains of a full-grown fœtus. The patient died of exhaustion and septic poisoning, derived from the decaying fœtus.

Moral—The ancient maxim, "a meddlesome midwifery is bad," should not be allowed to frighten us from the discharge of a manifest duty.

Dr. Beech, president of the Southern Michigan Medical Society, in his address before that body, in speaking of heterologous tumors, praises the deobstruent powers of conium, and remarks: "I should not excuse myself for opening this subject did I not express a firm conviction of the propriety of extirpating scirrhus and encephaloid masses *whenever*, *wherever*, and as *often soever* as they can be reached. * * There is an honest hope of relief for a great majority, and a certainty of cure for some, if you will not drive them to despair."

Dr. Currey, in the *Virginia Medical Monthly*, advocates the return of physicians to the old practice of dispensing their own medicines from their offices, and urges many reasons why this should be done.

Mr. C. Lewis Diehl states, in the *American Practitioner*, that the leaves of India senna yield to strong alcohol the griping and bitter principles of the senna, but not its purgative principle. He suggests a fluid extract to be made with aqueous menstrunum, after the leaves have been previously exhausted by strong alcohol. Such extract has but little taste and does not gripe. It is in use in Louisville, and meets with favor from the profession.

Dr. L. P. Yandell, Jr., reports his observations of the comparative effects of the sulphates of cinchonia and of cinchonidia: "Sulphate of cinchonidia is less bitter than sulphate of quinia, but more bitter than sulphate of cinchonia, and produces as much noise in the head as sulphate of quinia. Headache, sense of heat, and fulness about the head, disturbance of stomach, and dryness of mouth and throat are as frequent and decided from it as from sulphate of cinchonia. As a tonic it is not superior to the other alkaloids. * * In febrifuge virtue it is less than half as potent as sulphate of quinia, and a third less efficacious than sulphate of cinchonia. * * It has all the drawbacks attaching to sulphate of cinchonia, and no superiority in any respect over it, while it is nearly thrice its price."

These observations differ in result from those of some other experimenters, who assert for the cinchonidia a full equality with quinia, and great superiority over cinchonia.

In the December of the *American Practitioner* we find an extract from a report of Dr. D. W. Yandell to the Kentucky State Medical Society, on ovariotomy, in which he gives the statistics of this operation in the city of Louisville—the operation having been done thirty-six times, with nine recoveries and twenty-seven deaths.

A colored woman at the Columbia Hospital for Women has been treated for diabetes mellitus with salicylic acid in pill, using ten grains thrice daily. She had double, diabetic cataract, and there was fourteen per cent of sugar in the urine. Under the influence of the salicylic acid the sugar disappeared from the urine, and all other symptoms were correspondingly relieved.

*The American Medical Weekly* recommends that physicians return again to the time-honored practice of dispensing their own prescriptions to their patients.

In a lecture upon abortion (*Medical and Surgical Reporter*) Prof. Lusk urges the importance of emptying the uterus completely in all cases. He even insists upon this in cases which have been neglected one, two or three weeks, and the retained ovum is decomposed. He condemns the use of placenta forceps, and advises the finger to be introduced into the uterus, whilst the organ is pressed well down within reach by a hand upon the hypogaster.

Dr. Steele, in the *Pacific Medical and Surgical Journal*, commends the Grindelia Robusta as a specific in poisoning by rhus toxicodendron. It is applied locally in form of hydro-alcoholic fluid extract. He also employs it internally in asthma, rose cold and hay fever, in dose of ten to twenty drops every half hour until relief is obtained.

The *Pacific* defines the regulation of prostitution by law to be "A law for the protection of men from the diseases of women, and the exposure of women to the diseases of men; a law to provide places and salaries for male inspectors and for police officers; a law to furnish young men as well as married men with the means of gratifying their lust with impunity; a law to lower the standard of public morals, to discourage matrimony, to diminish the growth of population, and to inoculate the American republic thoroughly with the customs and vices of the old world."

Prof. Simon, of Heidelburg, has recently devised a method of exploring the ureter in women by means of both a probe and a small catheter, introduced even as far as the pelvis of the kidney. He first dilates the urethra so as to admit of the introduction of the index finger into the bladder, by which the probe or catheter is guided into the orifice of the ureter. In adults the urethra may be dilated to the extent of allowing exploration of the interior of the bladder with the finger without destroying its sphincter action. By his method the interior of the viscus may be fully and most satisfactorily explored.

THE MEDICAL ASSCCIATION OF GEORGIA will convene in its 27th Annual Session, in Augusta, April 19th. The railroads will pass members at half rates. DESAUSSURE FORD, President.

J. T. JOHNSON, Secretary.

